# Irathérapie dans la maladie de Basedow: place et efficacité

**DOI:** 10.11604/pamj.2020.36.341.21623

**Published:** 2020-08-25

**Authors:** Ali Sellem, Wassim Elajmi, Rania Ben Mhamed, Nesrine Oueslati, Haroun Ouertani, Hatem Hammami

**Affiliations:** 1 Service de Médecine Nucléaire, Hôpital Militaire Principal d´Instruction de Tunis, Tunis, Tunisie,; 2 Service d´Endocrinologie, Hôpital Militaire Principal d´Instruction de Tunis, Tunisie,; 3 Service d´Oto-Rhino-Laryngologie. Hôpital Militaire Principal d´Instruction de Tunis, Tunisie

**Keywords:** Hyperthyroïdie, Basedow, maladie iode radioactif, Hyperthyroidism, Grave’s disease, radioactive iodine disease

## Abstract

Le traitement de la maladie de Basedow repose sur trois thérapies: le traitement médical par les antithyroïdiens de synthèse, la chirurgie et l´irathérapie. L´objectif de notre étude était d´étudier la place et l´efficacité du traitement à l´iode radioactif dans le traitement de la maladie de Basedow. Une étude rétrospective portant sur 54 patients suivis pour une maladie de Basedow et traités par iode 131. On a mené en une étude descriptive des aspects épidémiologiques, cliniques, para cliniques et thérapeutiques et du le taux de rémission à court et à moyen terme. Le sex-ratio était de 0,45. L´âge moyen est de 38,33 ± 12,7 ans. Les signes fonctionnels les plus fréquemment retrouvés étaient l´amaigrissement, les tremblements et les palpitations. La FT4 moyenne est de 54,51 ± 19,56ng/dl (extrêmes: 8,90-100). La TSHus moyenne de nos patients était égale 0,074 ± 0,29 µUI/ml. Les antithyroïdiens de synthèse ont été prescrits chez 49 patients avec une persistance d´une hyperthyroïdie dans 83,67% des cas. L´irathérapie a été prescrite en première intention dans 9,3% et en 2ème intention dans 90,7% des cas. L´activité moyenne était égale à 13,29 mCi±1,46 avec des extrêmes allant de 10 à 15 mCi. Le premier contrôle hormonal post-irathérapie, réalisé après un délai moyen de 1,91 mois, a montré une rémission (eu- ou hypo-thyroïdie) chez 29 patients soit 53,7%. Après 12 mois de suivi, l´évolution était marquée par une rémission dans 88,88% (euthyroïdie chez 14,8% et l´hypothyroïdie chez 74%). L´irathérapie est un traitement efficace de la maladie de Basedow. Une dose forfaitaire forte d´iode radioactif permet d´obtenir un taux de rémission élevé.

## Abstract (en)

The treatment of Graves’ disease is based on three therapies: medical treatment with synthetic antithyroid agents, surgery and radioactive-iodine therapy. The purpose of our study was to study the role and effectiveness of radioactive-iodine therapy for the treatment of Graves’ disease. We conducted a retrospective, descriptive study of the epidemiological, clinical, paralclinical and therapeutic features of 54 patients with Graves’ disease managed and treated with iodine-131 as well as of their short- and medium-term remission rate. The sex ratio was 0.45. The average age of patients was 38,33 ± 12.7 years. The most common functional signs were weight loss, tremors and palpitations. Mean FT4 was 54,51 ± 19,56 ng/dl (ranging from 8,90 and 100). Mean TSHus was 0,074 ± 0,29 µIU/ml. Synthetic antithyroid drugs were used in 49 patients; 83,67% of cases had persistent hyperthyroidism. Radioactive-iodine therapy was used as first-line therapy in 9,3% of cases and as second-line therapy in 90,7% of cases. Mean activity was 13,29 mCi ± 1,46 ranging from 10 to 15 mCi. The first assessment of hormonal status was performed after an average post-treatment period of 1,91 months; 29 patients (53,7%) achieved remission (eu- or hypo-thyroidism). After a 12 month-follow-up, patients’ course was marked by remission in 88,88% of cases (euthyroidism in 14,8% and hypothyroidism in 74% of cases). Radioactive-iodine therapy is an effective treatment for Graves’ disease. High radioactive iodine dose provides high remission rate.

## Introduction

La maladie de Basedow (MB) est la cause la plus fréquente d´hyperthyroïdie [1]. Ses complications multiples (cardiaques, neuromusculaires, osseuses) peuvent être graves et justifient l´intérêt que suscite le traitement de cette affection [2]. Il existe trois armes principales thérapeutiques qui sont le traitement médical par antithyroïdiens de synthèse (ATS), le traitement chirurgical et le traitement par l´iode radioactif [2, 3]. Cette pluralité thérapeutique implique un choix pour chaque patient [4]. Notre étude a pour objectif d´étudier la place et l´efficacité du traitement à l´iode radioactif dans le traitement de la maladie de Basedow.

## Méthodes

Il s´agit d´une étude rétrospective, descriptive, monocentrique et transversale portant sur 54 patients suivis pour une maladie de Basedow et adressés au service de médecine nucléaire du pour un traitement à l´iode 131. Ont été inclus les patients diagnostiqués comme porteurs d´une maladie de Basedow et qui ont bénéficié d´un traitement par l´iode 131 pour la première fois de leur vie. Pour tous les patients, nous avons noté les données épidémiologiques, leurs antécédents personnels et leurs antécédents familiaux, leurs données cliniques et paracliniques: les dosages hormonaux (TSH et de FT4), les dosages immunologiques (Anticorps anti-récepteurs à la TSH et les autres anticorps antithyroïdiens anti peroxydase et anti-thyroglobuline) et les données de l´imagerie (échographie cervicale et scintigraphie).

L´évolution sous ATS (Basdène) se faisait soit vers une rémission ou un échec définit par la persistance d´une hyperthyroïdie après un traitement à pleine dose (Basdène 5mg à raison de 16 comprimés/jour) pendant 2 mois ou une impossibilité de dégresser les ATS (récidive de l´hyperthyroïdie) ou une intolérance (à type d´éruption, de cytolyse et d´agranulocytose). L´iode 131 était administré par voie orale après un sevrage de des ATS. Il a été indiqué en première intention: en raison d´une contre-indication aux ATS ou en deuxième intention après une préparation par les ATS.

L´évolution sous irathérapie a été faite à court terme (après un délai moyen de 2 mois) et à long terme après un délai de 12 mois et les patients étaient classés soit en rémission (en euthyroïdie ou en hypothyroïdie) soit en échec de traitement avec la persistance de l´hyperthyroïdie. Les données ont été saisies et analysées au moyen du logiciel IBM SPSS Statistics version 15. Les comparaisons entre les variables quantitatives sont faites par le test d´analyse de variance ou le test de Student et en cas de faibles effectifs par le test non paramétrique de Mann et Whitney. Les comparaisons entre les variables qualitatives sont faites par le test de Chi-deux (χ^2^) de Pearson et en cas de non validité de ce test par le test exact bilatéral de Fisher.long terme au bout de 12 mois.

## Résultats

Dans notre série, il existe une nette prédominance féminine, avec un sexe ratio de 0,45. L´âge moyen des patients est de 38,33 ± 12,7 ans avec des extrêmes allant de 20 à 79 ans et une médiane à 36,0 ans. 59% des patients ont un âge inférieur à 40 ans. Nous avons comparé l´âge des patients en fonction du sexe. Chez les patientes l´âge moyen est de 40,29 ± 15,2 et une médiane à 39 ans alors que chez les patients de sexe masculin l´âge moyen est de 37,43 ± 11,56 et une médiane à 36 ans. L´étude statistique a conclu qu´il n´y avait pas de différence significative entre les femmes et les hommes en ce qui concerne l´âge (p=0,350). Seulement sept patients étaient tabagiques soit 13%, tous étaient de sexe masculin.

Six patients, soit 11,11%, ont une maladie auto-immune associée dont un diabète de type 1 chez deux patientes. Chez quatorze patients (25,9%), nous avons retrouvé des antécédents familiaux. Les signes cliniques les plus fréquemment rapportés par nos patients étaient respectivement l´amaigrissement (72,2% des cas), les tremblements des extrémités (63% des cas) et les palpitations (48,1% des cas). Un goitre a été retrouvé chez 34 patients soit 63% des cas. Les stades les plus fréquemment retrouvés étaient les stades 1b et 2 avec un nombre total de 27 patients sur les 34 patients (79,4%) qui présentent un goitre. Une exophtalmie a été observée chez 18 patients (33,3%), elle était minime chez 10 patients, modérée chez 6 patients et sévère chez 2 patients. Elle est unilatérale chez 14 patients et bilatérale chez 4 patients. Des signes palpébraux ont été associés à l´exophtalmie chez 6 patients. Une cardiothyréose à type d´arythmie complète par fibrillation auriculaire (ACFA) est présente chez 6 patients (11,1%).

La FT4 moyenne est de 54,51 ± 19,56ng/dl (extrêmes: 8,90-100). La TSHus de nos patients variait entre 0,01 et 2 avec une moyenne égale à 0,074 ± 0,29 µUI/ml. Les anticorps anti thyroglobulines sont positifs (>35UI/ml) chez 63,36% des patients. La valeur moyenne des anticorps anti thyroglobuline était de 322, 85UI/ml avec des extrêmes allant de 20 à 3000UI/ml. Les anticorps anti peroxydases sont positifs (>40UI/ml) chez 46,1%. La valeur moyenne des anticorps anti peroxydases était de 307,31UI/ml avec des extrêmes allant de 10 à 3000UI/ml. Ils ont été pratiqués chez seulement 29 patients (53,7%). Ils sont positifs dans tous les cas. Leur taux moyen est de 11,77 UI/l (extrêmes: 2-40). Une échographie cervicale a été réalisée chez 41 patients (79,25%) retrouvant un goitre hétérogène dans 39% des cas, un goitre nodulaire dans 22%, un goitre homogène dans 17,1% des cas, une thyroïde de taille normale et hétérogène dans 14,6% des cas et une thyroïde de taille normale dans 7,3% des cas.

La scintigraphie thyroïdienne a été réalisée chez 49 patients soit 90,7%. Elle a montré une fixation intense au niveau de la glande thyroïdienne chez tous les patients. Il s´y est associé une zone hypofixante chez deux patients correspondant à un nodule froid. Les ATS ont été prescrits chez 49 patients sous forme de Benzyl-thiouracile (Basdène^®^) sous forme de comprimés à 25mg à la dose moyenne de 10,47 cp/j±1,85 cp/j avec des extrêmes allant de 6 à 12 cp/j. La durée moyenne du traitement médical était de 10,39 mois ± 13,03 mois avec des extrêmes allant de 2 jours à 75 mois. Chez trois patients (6,12%) le traitement médical a été arrêté durant la première semaine (respectivement 2, 3 et 6 jours) pour intolérance. L´évolution sous ATS a été marquée par la persistance d´une hyperthyroïdie chez 41 patients (83,67%) et l´obtention d´une euthyroïdie chez 8 patients (16,33%). La durée moyenne du traitement par les ATS était de 14,88 mois ± 12,69 mois avec des extrêmes allant de 1 mois et 40 mois pour les patients chez qui les ATS ont permis l´obtention d´une rémission. Chez les patients avec une hyperthyroïdie persistante sous ATS, La durée moyenne du traitement par les ATS était de 9,52 mois ± 13,06 mois avec des extrêmes allant de 2 jours à 75 mois.

**Figure 1:**
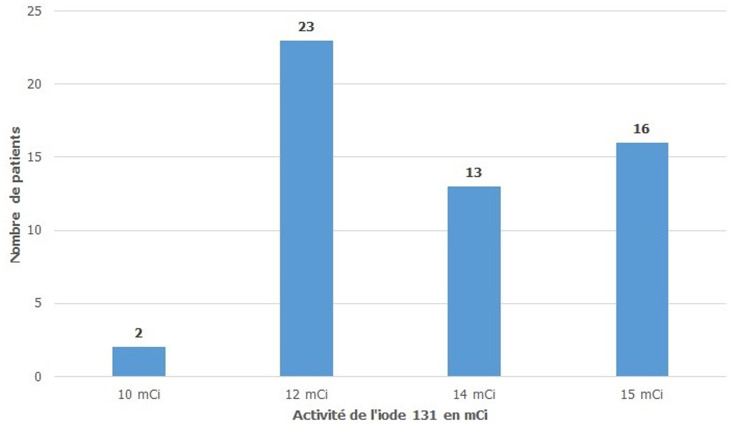
répartition des patients selon l´activité d´I131 reçue

**Figure 2:**
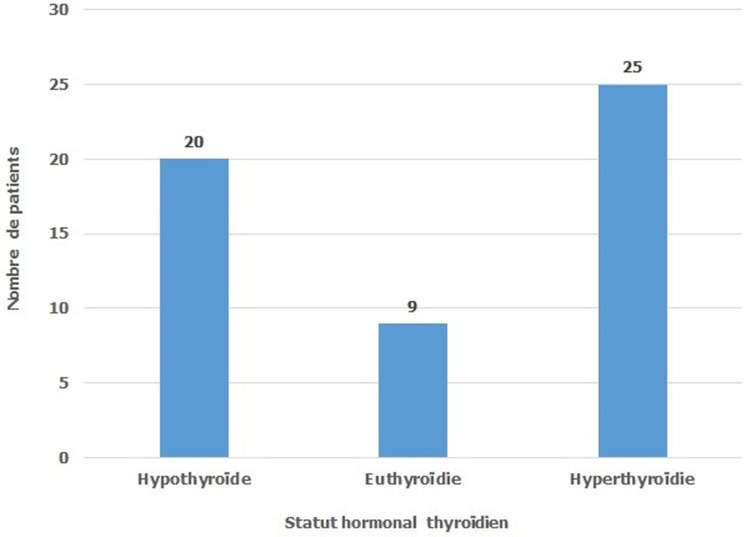
répartition des patients au 1^er^contrôle après un délai moyen de 1,91 mois

**Figure 3:**
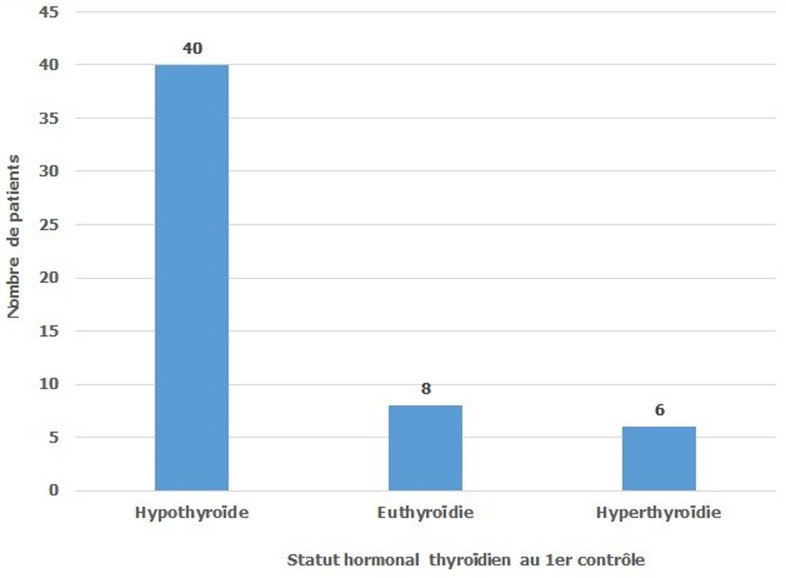
répartition des patients au 1^er^contrôle après 12 mois

Tous nos patients ont bénéficié d´une cure d´irathérapie en 1ère intention sans passage par un traitement médical chez 5 patients (9,3%) et en 2ème intention chez 49 patients (90,7%). Les ATS ont été arrêtés en moyenne 6 jours (extrêmes de 5 à 7) avant l´irathérapie. Une corticothérapie par voie orale, à la dose de 1mg/kg, a été indiquée chez 6 patients les deux patients ayant une exophtalmie sévère et quatre patients sur les six ayant une exophtalmie modérée. Tous nos patients ont bénéficié d´une 1ère dose d´iode 131. L´activité moyenne était égale à 13,29 mCi ± 1,46 avec des extrêmes allant de 10 à 15 mCi (Figure 1). L´évolution de l´exophtalmie a été marquée par une stabilisation chez les 8 patients qui ont bénéficié d´une corticothérapie préventive et qui présentaient une exophtalmie modérée chez 6 patients et une exophtalmie chez 2 patients. Par contre on a noté une aggravation de l´exophtalmie chez 2 patients parmi les 10 patients qui présentaient une exophtalmie minime et qui n´avaient pas bénéficié d´une corticothérapie préventive. On constate que la corticothérapie a un effet bénéfique sur l´évolution de l´exophtalmie (p=0,006). Le premier contrôle hormonal post-irathérapie, réalisé après un délai moyen de 1,91 mois avec des extrêmes allant de 1 et 4 mois, a montré que la cure d´iode 131 était efficace (avec l´obtention d´une eu- ou d´une hypo-thyroïdie) chez 29 patients soit 53,7% (Figure 2). Le deuxième contrôle hormonal réalisé 12 mois après l´irathérapie a montré que l´irathérapie a été efficace chez 88,88% et que parmi les 25 patients qui étaient en hyperthyroïdie lors du premier contrôle 6 seulement le sont restés (Figure 3). Aucune complication de l´irathérapie à court ou à moyen terme n´a été notée.

## Discussion

L´âge moyen des patients suivis pour maladie de Basedow et traités par l´iode 131 était de 37,3 ± 10,4 ans avec 31% âgés de moins de 30 ans dans l´étude de Yamashita Y *et al*. [5], de 49 ans (22 à 75 ans) [6]. Dans notre étude, l´âge moyen de nos patients était de 38,33 ± 12,7 ans, dont 28% ont un âge inférieur à 30 ans. Malgré que l´irathérapie soit un traitement de choix dans le traitement définitif de la maladie de Basedow, il persiste actuellement un manque de consensus concernant la dose thérapeutique optimale [7, 8]. En effet, il existe 2 protocoles différents l´un utilisant des activités standards fixes indépendantes des caractéristiques du patient et l´autre utilisant des activités calculées en fonction de différents paramètres (taille de la thyroïde, pourcentage de la fixation du radiotraceur au niveau de la thyroïde). Les études faites comparant ces différentes approches thérapeutiques ont donné des résultats contradictoires [9, 10]. Dans notre étude, nous avons opté pour l´activité standard. Selon les dernières recommandations de l´association américaine de la thyroïde (ATA), une dose fixe et suffisante d´iode 131 doit être administrée en une seule application. Cette dose est généralement comprise entre 10 et 15 mCi [11-13]. Nous avons respecté ces recommandations dans notre étude, en effet l´activité moyenne administrée est de 13,29 mCi ± 1,46. La seule contre-indication absolue à l´irathérapie est la grossesse [11, 14, 15], car l´iode 131 peut occasionner une hypothyroïdie fœtale. Il est recommandé de prévenir une conception durant au moins les 6 mois suivant la dose d´iode radioactif [16]. L´allaitement constitue aussi une contre-indication absolue à l´irathérapie à cause de son passage dans le lait maternel [15, 16]. Ces recommandations ont été respectées dans notre série.

Le prétraitement par les ATS avant l´irathérapie est généralement utilisé pour la déplétion des hormones thyroïdiennes et diminuer ainsi le risque d´exacerbation de l´hyperthyroïdie [11, 17]. Les inconvénients des ATS sont la toxicité hématologique et le risque élevé de récidive à l´arrêt du traitement (50%) d´où la nécessité d´une surveillance active et rapprochée [11, 17]. L´influence du prétraitement par les ATS sur l´efficacité de l´irathérapie est controversée. Malgré que certains auteurs rapportent une association des ATS avec un taux d´échec du traitement par iode radioactif plus important (ces études préconisent l´effet radio protecteur des ATS) [18], d´autres ne rapportent pas cette association [19, 20]. Walter *et al*. [21] a montré que pour obtenir un résultat optimal avec le traitement par IRA, les ATS doivent être arrêtés au minimum une semaine avant le traitement ablatif. Dans notre étude, les ATS ont été prescrits chez 49 patients (89%) et arrêtés en moyenne 6 jours avant l´irathérapie. La relation entre le traitement de l´hyperthyroïdie due à la maladie de Basedow et le cours d´évolution d´une ophtalmopathie Basedowienne associée est controversée [22]. Plusieurs études ont montré l´effet aggravant de l´irathérapie sur cette orbithopathie [23, 24]. Approximativement 15% des patients peuvent présenter après traitement par l´iode 131 l´apparition d´une ophtalmopathie ou l´aggravation d´une ophtalmopathie préexistante [25, 26]. Un traitement prophylactique par prednisone per os durant et après le traitement par l´iode 131 peut significativement diminuer le risque de développement ou d´aggravation d´ophtalmopathie [26, 27].

Dans notre étude, une corticothérapie (prednisone à la dose de 1mg/kg) a été prescrite chez 8 patients en raison d´une exophtalmie modérée à sévère avec une bonne évolution (stabilisation) à une année du traitement. Le succès d´un traitement par l´irathérapie d´une hyperthyroïdie se définit par l´obtention d´une euthyroïdie ou d´une hypothyroïdie. La persistance de l´hyperthyroïdie après traitement définie l´échec du traitement par l´iode 131. La plupart des auteurs considèrent actuellement l´hypothyroïdie comme un objectif thérapeutique [28-31]. Selon les données disponibles dans la littérature, le taux d´hypothyroïdie post irathérapie peut atteindre plus de 80% des patients à 12 mois du traitement. En effet, l´hypothyroïdie est notée chez: 86% des patients dans l´étude faite par Vija Racaru *et al.*[29], 58% des patients dans l´étude d´Isgoren *et al*. [18], 37.5% des patients dans l´étude de Husseni [32], et est rapportée chez 56.7% et 71.1% des patients ayant reçu respectivement 10 et 15 mCi d´iode 131 dans l´étude de Santos *et al*.[33]. Dans la plupart des études, la guérison dans la maladie de Basedow est obtenue dans plus de 80% des cas [3, 7, 8, 10, 29, 30, 33]. Le taux d´euthyroïdie après un an d´évolution post irathérapie est de 69% des cas dans l´étude de XU Jiehua *et al*. [34] et de 20% dans l´étude d´Isgoren *et al*. [35]. Dans notre étude, le taux de guérison à 12 mois de l´évolution est de 88.88%. Le taux de l´euthyroïdie est de 14.8% alors que le taux de l´hypothyroïdie est de 74%. L´irathérapie est généralement bien tolérée mais les complications les plus fréquemment décrites dans la littérature sont à type d´exacerbation de l´ophtalmopathie, une thyroïdite [16, 36]; rarement les patients éprouvent une cervicalgie et une augmentation du volume du goitre ce qui peut occasionner une obstruction des voies aériennes supérieures rapidement résolutive sous corticothérapie et une faible minorité des patients développe une exacerbation passagère de la thyrotoxicose [33, 37]. Dans notre série nous n´avons retrouvé aucune complication.

Le traitement de la maladie de Basedow repose actuellement sur trois modalités thérapeutiques: le traitement médical par les antithyroïdiens de synthèse (ATS), souvent utilisés en première intention [2]; la chirurgie et l´irathérapie [10]. L´utilisation de l´iode radioactif comme agent thérapeutique dans la maladie de Basedow a été rapporté pour la première fois en 1942 [11, 12] et il s´est avéré être un traitement simple, sans danger, efficace et économique [1]. Actuellement l´iode 131 est largement considéré comme le traitement de choix pour la plupart des patients ayant une hyperthyroïdie [14-17] particulièrement en rapport avec une maladie de Basedow vue son innocuité et sa fiabilité [18]. Bien que son indication reste différente d´une équipe à une autre et d´un pays à l´autre: l´iode radioactif est utilisé comme traitement initial dans 75% aux états unis [3, 16], en Europe et au Japon, la majorité des patients ayant une maladie de Basedow sont traités par les ATS en première intention [19, 20]. Le choix du traitement chirurgical est surtout réservé pour les patients présentant une exophtalmie sévère, l´existence concomitante d´un nodule thyroïdien suspect ou malin, un goitre volumineux (>80gr) et compressif, une intolérance au traitement par les ATS et qui ne désirent pas être traités par l´iode radioactif [2, 19, 15, 33, 38, 39]. En dehors de ces indications limitées à la chirurgie, le traitement ablatif de choix reste l´irathérapie [36].

## Conclusion

La maladie de Basedow, endocrinopathie fréquente aux complications potentiellement graves pose essentiellement un problème thérapeutique. Les ATS présentent un risque élevé de récidive et la chirurgie ne doit être réservée qu´à des indications bien définies. Le traitement à l´iode 131, simple peu agressif et peu coûteux, est considéré actuellement comme le traitement de choix de la maladie de Basedow.

### Etat des connaissances sur le sujet

- La maladie de Basedow est une endocrinopathie fréquente et ayant des complications potentiellement graves;
- Le traitement de la maladie de Basedow est basée sur 3 traitements: médical (antithyroïdiens de synthèse), la chirurgie (thyroïdectomie totale ou subtotale) et l´iode radioactif;
- L´iode radioactif est un traitement efficace de la maladie de Basedow sans effets indésirables notables.

### Contribution de notre étude à la connaissance

- Notre population inclut 54 patients avec un âge moyen de 38,33 ± 12,7 ans présentant une maladie de Basedow et traités par l´iode radioactif. C´est un nombre conséquent;
- Dans notre étude, nous avons utilisé des doses forfaitaires d´iode 131, avec une activité moyenne de 13,29 mCi ± 1,46, indépendantes de l´âge, du poids de la thyroïde et du pourcentage de fixation du radiotraceur au niveau de la thyroïde. L´irathérapie a été prescrite en première intention dans 9,3% et en 2ème intention dans 90,7% des cas;
- Dans notre étude, nous avons jugé l´efficacité de l´irathérapie avec un recul de douze mois et nous avons pu obtenir une rémission dans 88,88% (euthyroïdie chez 14,8% et l´hypothyroïdie chez 74%).
